# Miscellany

**Published:** 1888-06

**Authors:** 


### Miscellany.

A Strange Case of Stone in the Bladder.—It seems that followers of the Taoist religion, which is one of the three great religions of China, are in the habit of introducing short bougies into the penis at night, for the purpose, as they express it, of "keeping the paths of life open." Even the priests expose themselves and contract gonorrhœa, and, as a result, have strictures, but they do not think of one being the result of the other, but consider it some act of the gods or demons, so they keep these little bougies (generally of metal) in the penis at night, being careful to lie upon the side. They are sworn to secrecy as to this practice, but in dealing with these two cases, the secret was divulged, though very reluctantly. In both of these cases, the instrument used to keep the "paths of life open" was a short piece of ivory chop-stick, about two and one-half inches long, which had slipped into the bladder and became the foundation of a calculus. One had been in twenty years. There are, no doubt, other rites of these heathen religions which are as absurd and dangerous as the one referred to.—*Indiana Medical Journal.*

---

We have received an announcement of the Medical and Pharmaceutical Congress, to be held in Barcelona, Spain, September 9th to 15th next, at the time when the International Exposition is also in session at the same place. Physicians from all parts of the world are invited to participate. Papers may be presented in any language, but must be accompanied by a summary in Spanish. Physicians contemplating a European visit this summer, will do well to take advantage of this opportunity to acquaint themselves with the condition of medical affairs in Spain. Those desiring to attend should address Dr. Rodriguez Y. Mendez, editor of *La Gaceta Medica Catalana.*

---

Dr. W. H. L. Hale, of Philadelphia, regards bismuth salicylate as a very valuable remedy in the treatment of bowel troubles. He prescribes it in cholera morbus, dysentery, the diarrhœa of typhoid fever, and tuberculosis, and also in dyspepsia, where there are acid eructations, heart-burn, etc. To an adult he gives fifteen to thirty grains, frequently combining it with bismuth subnitrate. He finds the remedy useful also as a local application for obstinate pruritus.

---

We have received from Frank S. Billings, Director of the Patho-Biological Laboratory of Nebraska, an elaborate report of his investigations regarding the nature of the Southern cattle plague, Texas fever. The author has done excellent work, and is certainly honest, if not accurate, in his conclusion that he has discovered the true germ of the disease. His comparison of the cattle plague with yellow fever will be of special interest to physicians.

---

Dr. Wm. Tremaine, U. S. A., has recently returned from his California trip much improved in health. He intends to reside in Buffalo, and devote himself exclusively to surgical practice.

A FUND of $10,000 has been offered by the family of Cabot, of Boston, the income of which is to be used as a salary for the Pathologist of the Massachusetts General Hospital. Dr. W. F. Whitney, of the medical school, has accepted the position, with title of Assistant Pathologist.

---

DR. WILLIAM S. BRYANT, of Boston, claims to have discovered that, in the majority of young infants, the portal and mesenteric veins are provided with valves. The discovery is only of interest in connection with the study of development.

---

A RUSSIAN chemist has discovered that many of the preparations of cod liver oil and olive oil in his country are adulterated with coal oil products. Some preparations contain as high as fifty per cent. of the coal oil, without affecting the taste or appearance.

---

THE excessive secretion of gastric juice has been designated *gastrosuccorrhœa*, in which the Latin *succus* is unfortunately sandwiched in between two Greek words. It would have been quite as easy to form a pure Greek derivative like *gastrochylorrhœa*.

---

THE Buffalo Medical and Surgical Association has elected the following officers for the ensuing year: President, Dr. P. W. Van Peyma; Vice-President, Dr. A. A. Hubbell; Secretary, Dr. Trowbridge; Treasurer, Dr. F. E. L. Brecht.

---

MR. T. B. CURLING, well known for his work on the "Testis and Rectum," died at Cannes, in March. He discovered the association of burns on the skin with ulcers of the duodenum.

---

DR. WM. C. BAILEY, of Albion, who has been a frequent contributor to the JOURNAL, was recently elected President of the Central New York Medical Society.

---

DAROZIEZ has demonstrated the existence of a sphincter surrounding the framen ovale in the fœtal heart.

THERE are six claimants to the discovery of the cancer bacillus. Germany has two, Italy two, France one, and Brazil one.

---

IT is said that Sir Morell Mackenzie's London practice is worth $75,000 a year. He often sees from sixty to seventy patients a day.

---

CHARITY HOSPITAL, New Orleans, treats 25,000 people yearly. As a teaching hospital, it has no superior, and few, if any, equals in the United States.

